# Ligninolytic characteristics of *Pleurotus ostreatus* cultivated in cotton stalk media

**DOI:** 10.3389/fmicb.2022.1035040

**Published:** 2022-11-23

**Authors:** Guoqing Li, Yahui Wang, Deshui Yu, Peilei Zhu, Guiyun Zhao, Caiyu Liu, Hongyuan Zhao

**Affiliations:** ^1^State Key Laboratory of Horticultural Crop Germplasm Resources Creation and Utilization of Ministry of Agriculture and Rural Affairs, Institute of Horticulture Research, Anhui Academy of Agricultural Sciences, Hefei, China; ^2^College of Life Science, Anhui Agricultural University, Hefei, China; ^3^Provincial Resource Database of Wood Rot Edible Mushrooms in Anhui Province, Hefei, China

**Keywords:** *Pleurotus ostreatus*, cotton stalk, lignin, ^1^H-NMR, FTIR, GC–MS

## Abstract

Biodelignification is widely regarded as a low-efficiency process because it is usually slow and difficult to control. To improve its efficiency and understand its mechanism, the present study analyzed the delignification characteristics of *Pleurotus ostreatus* grown on a cotton stalk medium. The results demonstrated that all strains of *P. ostreatus* can selectively degrade the cotton stalk lignin. When cultured in a cotton stalk medium for 60 days, *P. ostreatus* degraded lignin primarily during its mycelium growth with up to 54.04% lignin degradation and produced laccase and manganese dependent peroxidase with high activity levels at the peaks of 70.17 U/ml and 62.39 U/ml, respectively, but no detectable lignin peroxidase. The results of nuclear magnetic resonance spectroscopy and Fourier transform infrared spectroscopy analyses of significant changes in lignin structure revealed that syringyl (S) lignin units were more degraded than guaiacyl (G) lignin units, with a significantly elevated G/S ratio. The Gas Chromatography-Mass Spectrometer analysis of low-molecular-weight compounds revealed that the delignification resulted in the formation of alcohols, organic acids, benzodiazepines, and alkanes. Identified benzodiazepines implied the degradation of G and S units of lignin. These findings will help to improve the efficiency of biodelignification and expand our understanding of its mechanism.

## Introduction

Cotton stalks are a by-product of cotton planting process, and approximately 40 million tons of cotton stalks are produced annually throughout the cotton growing areas in China (Li and Zhang, 2016; Wang and Tuohedi, 2020). Cotton stalks typically contain 45% cellulose, 20% hemicellulose, 21% lignin, and a low percentage of ash (Akpinar et al., 2011; Li et al., 2014; Keshav et al., 2021). Cotton stalks are less expensive than other lignocellulosic materials and are therefore a highly desirable lignocellulosic material. However, lignocellulosic material contains lignin, a highly irregular and insoluble polymer that is covalently linked with hemicellulose and cellulose, making it difficult to be directly utilized in the biological transformation process (Xing et al., 2018; Li et al., 2021).

Compared with numerous physical and chemical delignification methods, biodelignification has several advantages such as mild reaction conditions, high product yields, less energy consumption, and low toxicity (Li and Zhang, 2014). White-rot fungi are the most effective microorganisms for removing lignin from lignocellulosic materials because they secrete lignin-degrading enzymes that catalyze the mineralization of lignin into CO_2_ (Ahmad et al., 2016; Rouches et al., 2016; Wang et al., 2019a). The ability to produce ligninolytic enzymes of two fungal cultures *Pleurotus ostreatus* PAU03 and *Phanerochaete chrysosporium* MTCC 787 were investigated, *P. ostreatus* and *Ph. chrysosporium* consortium pretreated wheat straw, rice straw, corn straw and corn cob with 80.3, 73.4, 76.6, and 73% delignification, respectively (Gill et al., 2022). Currently, *P. ostreatus* is the second largest cultivated mushroom in the world, second only to *Agaricus bisporus*, because of its ease of growth on various lignocellulosic materials, such as wood, sawdust, and wheat straw (Fernandez-Fueyo et al., 2016). Some researchers have discovered that certain varieties of *P. ostreatus* could selectively remove lignin from lignocellulose, and they believed that these varieties could be used for delignification in the conversion of lignocellulosic materials into value-added bioproducts such as ruminant feed and bioethanol (Dong et al., 2013; Salame et al., 2014; Suryadi et al., 2022).

There is, nonetheless, some controversies exist surrounding using *Pleurotus* spp. for delignification. Some *Pleurotus* spp. preferentially degrade cellulose in lignocellulosic materials, whereas others degrade more lignin than cellulose or hemicellulose in rice straws and wood bits, and still some others degrade both lignin and cellulose in bagasse in equal proportions (Javad et al., 2007; Dong et al., 2013). Therefore, whether *P. ostreatus* strains selectively degrade cotton stalk lignocellulose is unclear and requires further investigation. *Pleurotus* spp. are thought to degrade lignin with laccases (LaCs) and manganese dependent peroxidases (MnPs) that they produce (Knop et al., 2014; Xiao et al., 2017; Wang et al., 2019b). However, whether they can produce lignin peroxidases (LiPs) is debatable and has yet to be proven (Asgher et al., 2016). Moreover, the degradability of lignin by using white-rot fungi depends on its content, composition, and structure. *Ceriporiopsis subvermispora* preferentially degrades the syringyl (S) unit of lignin in genetically-modified poplar (*Populus simoni* × *Populus Nigra*) (S, 83.3%), as opposed to the guaiacyl (G) lignin unit of wild poplar (G, 66.7%) (Skyba et al., 2013). Certain *P. ostreatus* strains preferentially degrade the S unit of sugarcane lignin (a type of monocotyledon lignin) (Dong et al., 2013); consequently, whether they can degrade cotton lignin (a kind of dicotyledon lignin) in the same manner requires further investigation.

To investigate whether *P. ostreatus* degrades cotton stalk lignin and what is their role in degrading cotton stalk lignin, growth tests of *P. ostreatus* on cotton straw medium were conducted and the strains with high selectivity factor and strong ability to degrade lignin were selected as well as the ligninolytic characteristics of the strains inoculated in cotton stalk medium were analyzed. At various stages following inoculation, the content of cotton stalk lignin (also known as Klason lignin) was assayed with the sulfuric acid hydrolysis method, whereas the structural changes in lignin polymer and its degradation products were characterized with Fourier transform infrared spectroscopy (FTIR), nuclear magnetic resonance spectroscopy (^1^H-NMR), and Gas Chromatography-Mass Spectrometer (GC–MS). The findings have guiding significance in improving the efficiency of lignin removal through biodegradation, promoting straw-based cultivation of edible fungi and lignocellulose recycling, providing information about the structural changes in lignin and its degradation products, and helping to expound the mechanism of lignin degradation by using white-rot fungi in nature.

## Materials and methods

### Fungal strains and inoculation

Eight commercial *Pleurotus ostreatus* strains, as shown in Table 1, were used in the experiment. These strains were preserved on potato dextrose agar (PDA, boiled juice of 200 g L^−1^ potato, 20.0 g L^−1^ glucose, and 15.0 g L^−1^ agar added to solid medium) plates at 4°C. To prepare the inoculate, the mycelium agar plugs with 5 mm in diameter inoculated in 250 ml tissue bottles with cotton stalk solid medium (5 g dry cotton stalk powder with 60 mesh sieves added 22 ml special culture solution, which contains ammonium tartrate 22.0 g L^−1^, KH_2_PO_4_ 20 g L^−1^, MgSO_4_·7H_2_O 13.8 g L^−1^, CaCl_2_1.0 g L^−1^, NaCl 0.6 g L^−1^, MnSO_4_·H_2_O 0.35 g L^−1^, FeSO_4_·7H_2_O 60 mg L^−1^, CoCl_2_·6H_2_O 110 mg L^−1^, ZnSO_4_·7H_2_O 60 mg L^−1^, CuSO_4_·5H_2_O 95 mg L^−1^, AlK(SO_4_)_2_·12H_2_O 6 mg L^−1^, H_3_BO_3_ 6 mg L^−1^, Na_2_MoO_4_·2H_2_O 6 mg L^−1^, and VB_1_ 100 mg L^−1^), were cut along the edge of the actively growing colonies, which had been cultivated on PDA plates for 7 days at 25°C.

**Table 1:** Eight *P. ostreatus* strains used in this study.

Stains	Sources
Wanping 1	Horticultural Research Institute of Anhui Academy of Agricultural Sciences
Zaoqiu	Institute of applied fungi, Huazhong Agricultural University
Heiping A	Institute of applied fungi, Huazhong Agricultural University
Suping 1	Vegetable Research Institute of Jiangsu Academy of Agricultural Sciences
Suping 3	Vegetable Research Institute of Jiangsu Academy of Agricultural Sciences
Xinping 400	Vegetable Research Institute of Jiangsu Academy of Agricultural Sciences
P17	Horticultural Research Institute of Anhui Academy of Agricultural Sciences
Tianda 300	College of forestry and landscape architecture, Anhui Agriculture University

### Determination of cotton stalks degradation

Add 50 ml of neutral detergent to 0.5 g of sample, followed by holding at 100°C for 1 h, filter and wash the sample with acetone aqueous solution, then add 50 ml of 2 M hydrochloric acid solution, hold at 100°C for 50 min, filter the filtrate to determine the hemicellulose content using lichen phenol colorimetric method. The residue was washed with water to neutral and washed twice with acetone, dried at 60°C, then placed in a 50 ml beaker, added 5 ml of 72% H_2_SO_4_, hydrolyzed at 20°C for 3 h, then added 45 ml of distilled water, room temperature overnight, the filtered filtrate was used to determine the cellulose content by a colorimetric method using anthrone reagent. The residue was then washed with water to pH 6.5 and the lignin content was obtained by weight of the residue dried at 80°C minus the weight of the ash after 550°C ashing.

Lignin (or cellulose, or hemicellulose) degrading rate (%) = [total lignin (or total cellulose, or total hemicellulose) degraded/total lignin (or total cellulose, or total hemicellulose) contained in the original sample] × 100; selectivity factor = (lignin degradation rate/cellulose degradation rate) × 100.

### Measurement of lignin degrading enzyme activity

The samples are removed every 5 days, added to 15 ml of purified water in batches, and extracted overnight under 4°C. Thereafter, vibrating extraction is performed at 200 r/min for 1 h, followed by refrigerated centrifugation at 12,000 r/min for 10 min. Finally, the supernatant was obtained as the enzyme solution. The LaC activity was determined at 25°C by 2,2-azinobis-(3-ethylbenzthiazoline-6-sulfonate) (ABTS) as substrate. The assay mixture contained 2 mM ABTS and 0.1 M sodium-citrate buffer, pH 3,0. Oxidation of ABTS was followed by absorbance increase at 420 nm (ɛ = 36,000 M^−1^ cm^−1^) for 1 min. One unit activity is defined as the amount of enzyme that transformed 1 μmol of substrate per min (Ya et al., 2013). Using hydrogen peroxide oxidation, the performance of MnP activity was examined (Lueangjaroenkit et al., 2019). The reaction system was 20 mmol L^−1^ tartrate-sodium tartrate buffer (pH 4.5) 0.8 ml, 2 mmol L^−1^ MnSO_4_ solution 0.05 ml, appropriate amount of enzyme solution, 2 mmol L^−1^ H_2_O_2_ 0.05 ml, and the change of absorbance value at 238 nm was measured per time unit. The absorbance value at 238 nm was measured and the change in the absorption spectrum of 0.01 was equal to one unit of enzyme activity. The activity of LiP activity was tested by oxidising veratryl alcohol. The reaction system was 1.85 ml of 0.24 mol L^−1^ sodium tartrate buffer (pH 3.0), 0.1 ml of 24 mmol L^−1^ quinacrine and an appropriate amount of enzyme solution. After preheating to 37°C, 0.05 ml of 6 mmol L^−1^ H_2_O_2_ was added to start the reaction, and the absorbance value was measured at 310 nm, and the change in optical density at 310 nm per minute was used as one LiP activity unit (Chandrasekaran et al., 2014). Throughout all the test, sterile water was used to maintain the blanks.

### Fourier transform infrared spectroscopy analysis

FTIR analysis was conducted to determine the functional groups that were converted during SCB degradation. The homogeneous and representative wafer samples obtained using KBr Pellets. The micro-FTIR spectra of local areas of the wafer specimen were measured using a NICOLET iN10 MX spectrometer (Thermos Nicolet Corporation, Madison, WI); connected to a Nicolet NicPlan IR microscope and an MCT detector. The spectral range was taken from 4,000 to 650 cm^−1^ at a resolution of 4 cm^−1^.

### Solid state ^1^H-NMR analysis

Lignin was extracted from the uncultured substrates and the substrates that were cultured with *P. ostreatus* after 30 and 60 days, respectively. A total of 20 mg of lignin samples was weighed and dissolved in 2 ml of pyridine: acetic anhydride (1:1) mixture. Nitrogen was charged into the reaction bulb, which was placed in the dark at room temperature for 72 h. After the reaction was completed, the reactant was dripped into diethyl ether until precipitate was formed, after which the solution was centrifuged to separate the precipitate. The precipitate was then washed with diethyl ether six to eight times to remove the pyridine odor from the precipitate. Finally, the completely acetylated lignin was obtained. The acetyl-treated lignin samples were dissolved in 0.5 ml of DMSO, with tetramethylsilane as the internal standard. Finally, the ^1^H-NMR assay was conducted with a Bruker-400 superconducting NMR spectrometer (Bruker, Switzerland) at a frequency of 400 MHz.

### GC–MS analysis of lignin-degrading products

A total of 0.1 g of stalk powders was weighed and added to 1.4 ml of cooled methyl alcohol. The container was then exposed to ultrasonic waves for 30 min and centrifuged for 10 min to remove the precipitate. Add equal volume of trichloromethane to the absorbed supernatant, vortex it for 1 min, and then centrifuge it at high speed. Thereafter, 1 ml of the supernatant is blow-dried with nitrogen. The sample without powders was used as the blank control. The cotton stalk powders that were not used to culture *P. ostreatus* served as the experimental control, whereas the cotton stalk powders that were used to culture *P. ostreatus* served as the experimental group. To each group, 60 μl of 20 mg mL^−1^ hydroxylamine hydrochloride-pyridine solution was added. The containers were then heated under 100°C for 2 h. Then, 60 μl of 20 mg mL^−1^ BSTFA+TMCS solution was added, and the containers. The container was again placed at 100°C for 2 h. Thereafter, the mixtures were subjected to analysis with a gas chromatograph-mass spectrometer (Agilent789A). Finally, the data were retrieved from the National Institute of Standards and Technology (NITS) spectral library.

### Statistical analysis

Three replicate trials were carried out for each sample, and all the experiments were repeated three times. Data were analyzed by one-way analysis of variance (ANOVA) using DPS 9.50 software, and expressed as the means ± standard deviations (SDs). Statistical significance was considered at the *p* < 0.05 level. All graphs were drawn with Excel 2010 software.

## Results

### Lignocellulose degradation and selectivity factors of *Pleurotus ostreatus* strains

**Figure 1:**
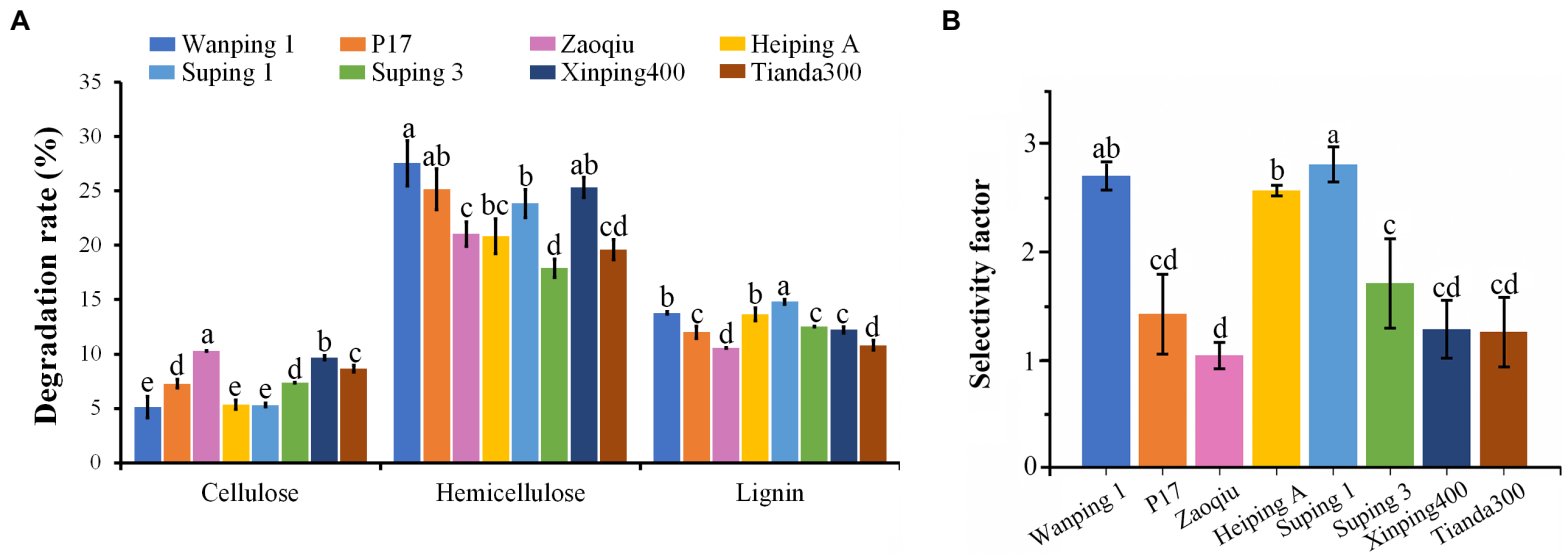
Degradation rates of three major components (cellulose, hemicellulose, and lignin) of cotton stalks using eight *P. ostreatus* strains and their selectivity factors. **(A)** Degradation rates of cotton stalks. **(B)** Selectivity factors of eight *P. ostreatus* strains. The different letters in the same pillars indicate significant differences according to the LSD at *p* ≤ 0.05 levels.

After 15 days following inoculation, the cotton stalk lignin-degrading capability varied greatly among the strains. Suping 1 achieved the highest lignin-degrading rate (14.82%), followed by Wanping 1 (13.79%) and Heiping A (13.65%) in that order. Zaoqiu and Tianda300 achieved the lowest lignin-degrading rate (10.57 and 10.81%, respectively), as well as P17, Suping3 and Xingping400 were at intermediate levels (Figure 1A). The degradation rate of hemicellulose was high for all strains ranging from 17.9 to 27.57%, with the lowest being Suping3. Furthermore, the cotton stalk lignin-degrading rates of the strains were higher than their cellulose-degrading rates (Figure 1A). During the 15-days inoculation, *P. ostreatus* preferentially degraded lignin in the cotton stalks, thereby displaying lignin-specific selectivity. The lignin degradation selectivity of the strains is expressed using the selectivity factor (Figure 1B). Among six strains, Zaoqiu achieved the lowest selectivity factor (1.03), whereas Wanping 1, Heiping A, and Suping 1 achieved higher selectivity factors (>2), and Suping 1 achieved the highest selectivity factor (2.80). Therefore, Zaoqiu and Suping 1 were selected for further study because of the large disparity between their selectivity factor and lignin-degrading rate.

### Varying lignin content in cotton stalks during degradation

**Figure 2:**
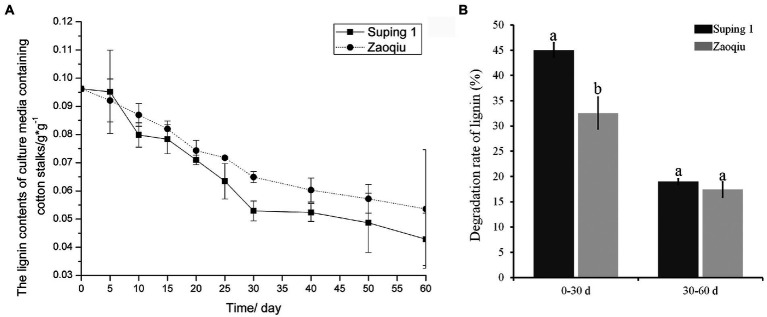
Dynamic monitoring of lignin content in cotton stalk medium inoculated with *P. ostreatus* strains (Suping 1 and Zaoqiu). **(A)** Changes of lignin content in cotton stalks medium after inoculation. **(B)** Degradation rates of cotton stalk lignin by Suping 1 and Zaoqiu at various stages. The different letters in the same pillars indicate significant differences according to the LSD at *p* ≤ 0.05 levels.

The lignin content of the cotton stalks degraded using the two selected strains (Zaoqiu and Suping 1) decreased gradually as the degradation continued. Moreover, the lignin content of cotton stalks in the medium inoculated with Suping 1 strain was lower than that of Zaoqiu from 25 to 40 days of incubation, which is the most productive stage of mycelial growth and the protoplast stage of fruiting body development (Figure 2A). The results demonstrated that *P. ostreatus* could decompose lignin and significantly remove lignin from cotton stalks. Suping 1 demonstrated significantly greater lignin degradation capability than Zaoqiu at the stage of fruiting body development, and such a remarkable difference may be caused by the specificity of the strain.

After the cotton stalks were degraded using the two strains for 30 days, the lignin content decreased rapidly in the first 30 days and then gradually in the following 30 days (Figure 2A). On day 30 following inoculation, the lignin-degrading rates of Suping 1 and Zaoqiu were 45.04 and 32.57%, respectively, apparently higher than those from the day 30 to day 60 (19.00 and 17.41%, respectively) (Figure 2B). These results suggest that biodegradation of lignin by *P. ostreatus* during growth on cotton stalks medium primarily happened in the mycelial growth stages.

### Extracellular LaC and MnP activities

**Figure 3:**
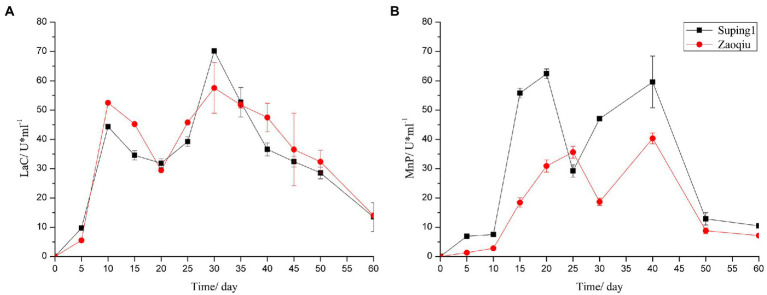
Ligninase enzyme activities of the cotton stalks degraded by *P. ostreatus* in solid-state fermentation. **(A)** Laccase; **(B)** Manganese peroxidase.

The LaC activity produced by Suping 1 and Zaoqiu began to increase rapidly on day 5 following inoculation, peaked at the 10th day after inoculation at 52.47 and 44.34 U mL^−1^, respectively, and then decreased. On day 20 following inoculation, the LaC activity levels of Suping 1 and Zaoqiu decreased to 29.41 and 31.85 U mL^−1^, respectively, and then LaC activity of Suping 1 and Zaoqiu started to increase again, reaching a maximum of 70.17 and 57.55 U mL^−1^, respectively, on day 30 after inoculation. Subsequently, the Lac activity of Suping 1 and Zaoqiu gradually decreased from 30 to 60 days of inoculation (Figure 3A). Similarly, the first peaks of MnP activity of Suping 1 and Zaoqiu was observed at day 20 and day 30 after inoculation with values of 62.39 and 35.61 U mL^−1^, respectively. The second MnP activity peaks of both Suping 1 and Zaoqiu was observed at the 40th day following inoculation with values of 59.59 and 40.33 U mL^−1^, respectively (Figure 3B). Furthermore, the MnP activity of Suping 1 was generally greater than that of Zaoqiu at 60 days after inoculation except for day 25, which may explain the stronger lignin degradation ability of Suping 1 than Zaoqiu.

### FTIR analysis

**Figure 4:**
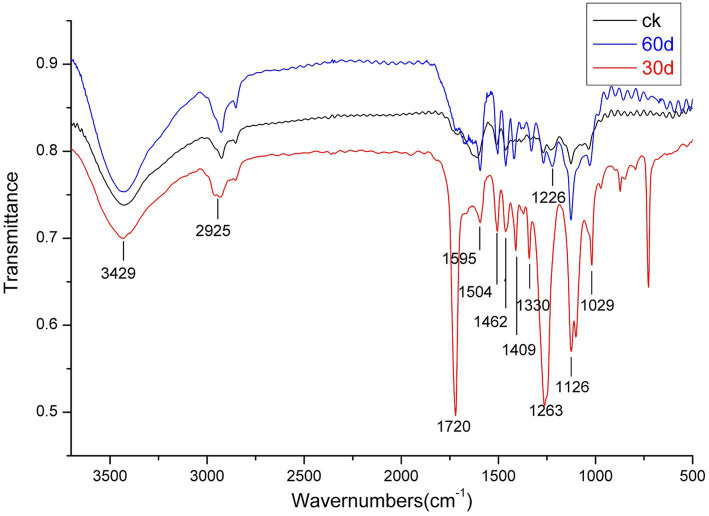
Infrared spectroscopy of milled wood lignin (MWL) from the cotton stalks pretreated by Suping 1 strain at various stages. ck stands for uninoculated cotton stalks medium; the red line represents 30 days pretreatment of cotton stalks medium; the blue line represents 60 days pretreatment of cotton stalks medium.

Because Suping 1 demonstrated a significantly greater lignin degradation capability than Zaoqiu, it was selected as the representative strain to study the modification of lignin structure and the small-molecular-weight metabolite produced using *P. ostreatus* during lignin removal. Figure 4 shows the results of an infrared spectroscopy analysis on the milled wood lignin (MWL) of the cotton stalks degraded using Suping 1.

A broad and prominent peak was observed in the region between 3,425 and 3,431 cm^−1^, which is the stretching vibration peak of the hydrogen bond in-OH. Furthermore, a C-H stretching vibration peak was observed in the region between 2,925 and 2,933 cm^−1^ (Cai et al., 2010). The characteristic absorption peaks of the functional groups of lignin are primarily concentrated in the fingerprint region between 1800 and 800 cm^−1^. Table 2 details the representative characteristic peak in the region between 4,000 and 400 cm^−1^. The functional group change during lignin degradation was studied by comparing the intensities of MWL’s characteristic absorption peaks at various growth stages with those of the control. According to the method proposed by Jin et al. (2005), we calculated the intensities of the main infrared spectrum band of MWL at various growth stages, with 1,504 cm^−1^ as a reference (Table 2).

**Table 2:** Infrared spectrum analysis of cotton stalk lignin after degradation.

Peak/cm^−1^	Functional group stretching	Ai / A_1504_		
		ck	30 day	60 day
3,429	Stretching in hydroxyl groups	1.43	1.03	0.91
2,925	C-H stretching in methyl and methyene groups	1.1	0.84	1.07
1720	C=O stretching in unconjugated ketone	0.93	2.03	1.07
1,595	Aromatic skeletal vibrations plus C=O stretching	1.05	0.96	0.96
1,504	Aromatic skeletal vibrations	0.99	1	1
1,462	C-H deformation in methyl	1.04	1	0.97
1,422	Aromatic skeletal combined with C-H in-plane stretching	0.98		0.99
1,409	Aromatic skeletal combined with C-H in-plane stretching	0.97	1.1	
1,330	Condensation of guaiacyl unit and syringyl unit, syringyl unit, and CH2 bending stretching	0.97	0.97	1.01
1,263	G ring plus C=O stretching	1.05	1.96	0.99
1,226	Aromatic C-O stretching (S units)	1.04	1.21	0.98
1,126	Aromatic C-H in plane deformations	1.13	1.62	0.9
1,029	Aromatic C-H in-plane deformation plus C-O deform. In primary alcohols plus C=O stretching	1.02	1.02	0.85
	A1263/A1226	1	1.62	1.01

Within the infrared spectrum band, the absorption of methyl and methylene peaked at 2932 cm^−1^. This peak lowered on day 30 of the mycelium stage, compared with that of the control group, suggesting that methyl and methylene were broken during lignin degradation (Chen et al., 2015). The absorption of keto-carbonyl, acetic ester, or other esters peaked at 1720 cm^−1^. On day 30 following inoculation, the relative absorption intensity (A1725/A1504) of MWL was 2.03, whereas that of the control group was 0.93. These findings suggested that oxidation occurred during lignin degradation by using *P. ostreatus*, thus weakening the bond between lignin and hemicellulose (Dong et al., 2013).

The band near 1,263 cm^−1^ was the stretching vibration of the G unit and C=O stretching in lignin, which is the major structural unit of lignin, whereas the band near 1,226 cm^−1^ corresponds to the S unit and C-O stretching in lignin (Cai et al., 2010). The band near 1,126 cm^−1^ depicted the deformation vibration of the aromatic nucleus C-H of the G lignin unit on the plane. On day 30 following inoculation, the relative intensities of the absorption peaks (A1263/A1504, A1226 /A1504, and A1126/A1504) of MWL were 1.96, 1.21, and 1.62, respectively, which are higher than those in the control group (1.05, 1.04, and 1.13, respectively). During lignin degradation, the intensities of the absorption peaks at 1,263, 1,226, and 1,126 cm^−1^ increased, indicating that the connection bond between the lignin units broke under the action of enzymic oxidation and produced lignin monomer analogs.

According to Jin et al. (2013), the intensities of the absorption peaks at 1,263 and 1,226 cm^−1^ (A1263/A1226) can be used to express the relative amounts of G lignin units and S lignin units. On day 30 following inoculation, the value of A1263/A1226 was 1.62, apparently greater than that of the control group (1.00) (Table 2). Compared with the G lignin unit, the S lignin unit was preferentially degraded during lignin degradation.

### ^1^H-NMR analysis

**Figure 5:**
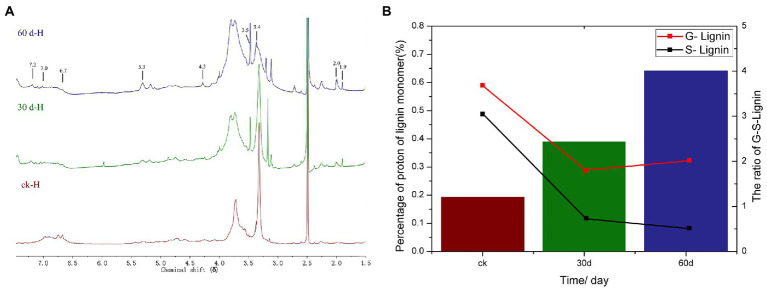
^1^H NMR analysis of cotton stalks control and degraded by *P. ostreatus* for 30 and 60 days. **(A)** ^1^H-NMR spectra of acetylated MWL from the cotton stalks at various degradation stages. **(B)** Percentage and ratio of G lignin units and S lignin units in acetylated MWL from the cotton stalks at various degradation stages.

The ^1^H-NMR spectrogram and results of the acetylated cotton stalk MWL at various periods are shown in Figure 5A and Table 3, respectively. Based on the ^1^H-NMR spectrogram for the acetylated lignin, the MWL range was distinguished, and MWL was integrated to calculate the proton ratio between functional groups and bond-type structures (Lu et al., 2008). In the ^1^H-NMR spectrogram for acetylated cotton stalk lignin at various degradation periods, signal peaks that appeared at the chemical shift point 7.3 × 10^−6^ – 6.8 × 10^−6^ represent the G lignin unit, whereas signal peaks that appeared at the chemical shift point 6.8 × 10^−6^ – 6.3 × 10^−6^ represent S lignin units.

**Table 3:** ^1^H-NMR analysis of acetylated milled wood lignin from the cotton stalk at various degradation stages.

Chemical shift region (δ)	Type of protons	Percentage of protons (%)		
		ck	30 day	60 day
7.3–6.8	Aromatic protons in guaiacyl units	0.59	0.29	0.32
6.8–6.3	Aromatic protons in syringyl units	0.49	0.12	0.08
6.3–5.6	Hα of β-O-4 and β-1 structures	0.01	0.03	0.2
5.6–5.2	Hα of β-5 structures	0.17	0.11	0.2
5.2–5.0	Hydrocarbon protons	0.17	0.1	0.16
4.9–4.5	Hα and Hβ of β-O-4 structures	0.51	0.38	0.38
4.5–4.2	Hα of β-β structures	0.29	0.18	0.46
4.2–3.0	H of methoxyl, methanol groups, and Hα in several structures	4.66	3.65	5.48
2.3–1.9	H of aromatic acetates	0.11	0.07	0.12
1.9–1.6	H of aliphstic acetates	0.13	0.08	0.13

The amounts of G lignin units and S lignin units in the cotton stalk varied at various degradation periods (t), as did the G:S ratio (Table 3; Figure 5B). On day 30 of degradation, the amount of G lignin units and S lignin units significantly decreased compared with those in the control group. On day 60 of degradation, the amount of G lignin units increased slowly and the amount of S lignin units decreased more slowly than on day 30 (Figure 5B). These results suggested that *P. ostreatus* degraded cotton stalk lignin primarily when its mycelium grew, which is consistent with the analysis of the lignin content of cotton stalk during the degradation. On day 60 of degradation, the amount of G lignin units increased that than on day 30, suggesting that *P. ostreatus* converted a part of S lignin units to G lignin units (Figure 5B). Furthermore, the amount of S lignin units decreased more significantly than that of G lignin units on day 30 of degradation, and the content ratio between G lignin units and S lignin units increased (Figure 5B) during the 60 days of degradation, indicating that *P. ostreatus* preferentially degraded the S lignin units of the cotton stalk lignin. These results are in agreement with the infrared spectroscopy analysis.

### GC–MS analysis

**Figure 6:**
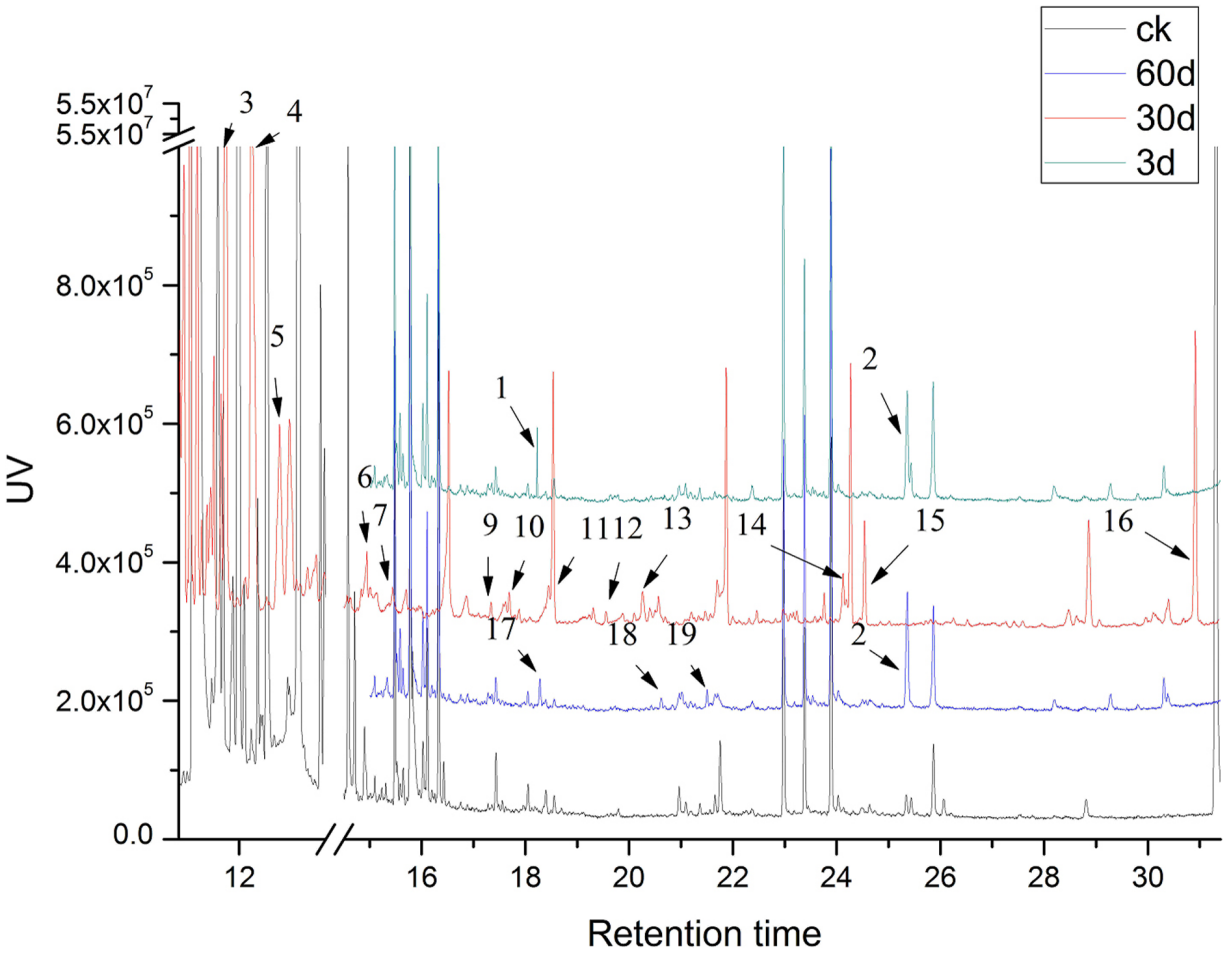
GC–MS analyses of lignin degradation compounds from samples collected 3, 30, and 60 days following inoculation.

The low-molecular-weight compounds were identified from the supernatant of cotton stalks inoculated with *P. ostreatus*. Apart from the identical compounds identified from the control group, other low-molecular-weight compounds are shown in Table 4 and Figure 6. These degradation products were categorized based on their chemical structure as alcohols, organic acids, benzodiazepines, and alkanes. The compounds identified from sample supernatants at varied stages varied substantially. On day 3, 30, and 60 after the inoculation of *P. ostreatus*, 2 (organic acid and benzodiazepines), 14 (organic acid, benzodiazepines, and alcohol), and 4 (organic acid, benzodiazepines, and alkane) low-molecular-weight compounds were, respectively, identified from the extracts of cotton stalk samples (Figure 6).

**Table 4:** Low-molecular-weight compounds identified as tetramethylsilane derivatives in chloroform extracts of *P. ostreatus* degraded cotton stalk samples at various growth stages.

No.^a^	Variety	Retention time (min)	Compounds
3	Alcohols	11.8	2,6-dimethyl-Cyclohexanol
5		12.5	Glycerol
2	Organic acids	25.3	9,12-Octadecadienoic acid
4		12.2	Carbamic acid
7		15.4	Ethanedioic acid
10		17.7	Azelaic acid
11		18.5	Tetradecanoic acid
13		20.3	n-Pentadecanoic acid
14		24.1	Linoleic acid
15		24.5	Octadecanoic acid
16		30.9	Hexadecanoic acid
1	Benzenes	18.2	Protocatechuic acid
6		14.9	2,6-Ditert-butylphenoxy
8		16.5	Thianthrene, 5,10-dioxide
9		17.3	Vanillic acid
12		19.6	Syringic acid
17		18.3	Benzoic acid
18		20.6	3,5-dimethoxy-benzoate
19	Alkanes	21.5	Dodecane

The similarity of each compound, which was obtained by comparison of MS with the NITS 2011 library, ranged from 80 to 99% . ^a^The numbers in the No. column represents peaks labeled in Figure 6.

In the preliminary experiment, we also detected the activity of the lignin-degrading enzyme in the cotton stalk samples on day 3 following inoculation. Therefore, we decided to collect a sample for GC–MS analysis on day 3 following inoculation. During the 3 days following inoculation, a key intermediate-protocatechuic acid-was detected in the extract of the degradation sample, but was not in the extract of the control group, suggesting that lignin degradation began on the third day following inoculation.

In addition to protocatechuic acid, vanillic acid and syringic acid were identified in the cotton stalk sample on day 30 following inoculation, and benzoic acid and 3,5-dimethoxybenzoic acid ester were identified on day 60 following inoculation. The production of vanillic acid and syringic acid was likely related to the oxidizing reaction between the two basic components of hard-wood lignin, sinapyl alcohol (G unit) and coniferyl alcohol (S unit) (Kumar et al., 2015). The general lignin-degrading mechanism is as follows (Chen et al., 2015): (1) The polyphenolic structure was formed through demethoxylation and hydroxylation followed by (2) the oxidation of the side chain of lignin monomer as well as the oxidation and falling off of the polyphenolic ring. During the cultivation of *P. ostreatus* on cotton stalks, aromatic compounds with benzene-ring structural units, such as vanillic acid, syringic acid, and benzoic acid, were produced. All of these substances had a structure similar to the lignin monomer, indicating that *P. ostreatus* was able to depolymerize lignin into a lignin monomer analog and further oxidize it to substances, such as organic acids and alkanes. That is, the production of these aromatic compounds, organic acids, and alkanes during biodegradation is consistent with the aforementioned degradation mechanism.

## Discussion

The research on cotton straw delignification is intended for application in industrial production and is mainly related to physical and chemical methods (Karthyani et al., 2020; Cheng et al., 2022; Dong Z. et al., 2022). With the intensive research on white rot fungi, some strains with high production of lignin degrading enzymes were also applied for delignification production (Meehnian et al., 2016; Wittner et al., 2021; Sabogal-Otalora et al., 2022). For example, Meehnian et al. (2017) used the white rot fungi *Daedalea flavida* MTCC 145 and *Phlebia radiata* MTCC 2791 to degrade cotton straw lignin to release glucose. The aim of our study was to investigate more efficient utilization of cotton straw cellulose after efficient lignin delignification by the strains and thus to obtain a higher conversion rate. The present study revealed that all eight strains were capable of preferentially degrading lignin, albeit to varying degrees of degradation. Wanping 1, Heiping A, and Suping1 had higher selectivity factors (>2) and higher lignin-degrading rates (>13%) in 15 days. Therefore, these three strains have the potential to be grown on stalks and to recycle lignocellulose. For instance, they can be used to convert lignocellulose into ruminant feed and as a pretreatment for delignification in the biomass energy industry (Salame et al., 2014). Early studies have revealed a positive correlation between the lignin-degrading rate and the fruiting body yield (Li et al., 2014). Therefore, these three edible fungi strains can also be used to increase yield in stalk-based cultivation.

When cultured for 60 days on cotton stalk solid medium, *P. ostreatus* produced highly active LaC and MnP, but no LiP. The *Daedalea flavida* MTCC 145 strain with high LiP and Lac activity and no MnP activity had high lignin degradation ability, the *Phlebia radiata* MTCC 2791 strain with high MnP activity and low Lac and Lip activity had weaker lignin degradation ability than the *D. flavida* MTCC 145, and the *Flavodon flavus* MTCC168 strain with low Lac and MnP and no Lip activity had extremely weak lignin degradation ability (Meehnian et al., 2017), which was attributed to the fact that after the initial depolymerization of lignin by peroxidase (LiP and MnP), lignolytic enzymes including laccase could easily penetrate into the cotton stalk cell wall, so the biodegradation of lignin requires the cooperation of multiple enzymes (Arora et al., 2002). The complex lignin-degrading enzyme system of white-rot fungi consists of extracellular enzymes such as LaC, MnP, and LiP. However, a specific type of white-rot fungus typically produces one, two, or three types of extracellular enzymes (Torres-Farrada et al., 2017; Han et al., 2021). *Phanerochaete chrysosporium* produces MnP and LiP, but not LaC (Coconi-Linares et al., 2015). *Pycnoporus cinnabarinus* does not produce LiP and MnP in its lignin-degrading-oriented culture, but it does produce a type of LaC isozyme (Henske et al., 2018). This study also reveals that *P. ostreatus* can produce LaC and MnP, but not LiP, which is consistent with previous studies (Ferreira et al., 2015; Zolciak, 2019).

The degradation of cotton stalk lignin by a CDs/CuO synergistic emulsion system was investigated by Dong Z. et al., (2022), and the results showed that H-type monomers were the main products of lignin degradation in this system. However, during lignin degradation by using *P. ostreatus*, S-type monomers and G-type monomers were the main degradation products, and the S lignin unit was more easily degraded than the G lignin unit, and the G/S ratio exhibited an upward trend. Thus, biodegradation of cotton stalk lignin differs from chemical degradation and the type, composition, and content of lignin affect its degradation by white-rot fungi. In the study by van Kuijk et al. (2016) changes in lignin composition were a direct result of lignin degradation since it was mainly related to the mechanisms of fungal degradation and less to substrate properties. In this study, 21% of the cotton stalk lignin belongs to the dicotyledon lignin (G-S). According to infrared spectroscopy and ^1^H-NMR analysis, the S lignin units are more susceptible to degradation by *P. ostreatus* than the G lignin units. However, for the bagasse lignin (25%), a kind of monocotyledon lignin (G-S-H), its S lignin is also preferentially degraded by *P. ostreatus* (Dong et al., 2013), suggesting that the preferential degradation of the S lignin units by *P. ostreatus* is unaffected by the type, content, and composition of stalk lignin. However, other studies have suggested that the type, composition, and type of stalk lignin influence the rate of lignin degradation by white-rot fungi (Skyba et al., 2013). Hence, in the recycling of stalks, we can select an appropriate white-rot fungus according to the variation in stalk lignin to shorten the treatment cycle and improve efficiency.

In the process of the degradation of cotton stalk lignin by *P. ostreatus*, seven types of aromatic metabolites were produced, including protocatechuic acid, 2,6-ditertbutyl phenoxy derivative, vanillic acid, syringic acid, benzoic acid, and 3,5-dimethoxy-benzoate. The production of these aromatic metabolites is indicative of the degradation of the G units and S units of lignin. The catabolic pathway of many lignin components produces vanillin or oxidizes vanillin to produce vanillic acid, which is then demethylated and converted into protocatechuic acid. After the oxidation ring of the relevant dioxygenase (such as 3,4-dioxygenase of protocatechuic acid) of fungus breaks, protocatechuic acid is degraded. All of these dioxygenases are the key enzymes of the β-ketoadipate pathway (Bugg et al., 2011; Martins et al., 2015; Li et al., 2022). Previous studies have demonstrated that the *P. ostreatus* mycelium, when induced by a lignin monomer analog (such as p-hydroxybenzoic acid), produces corresponding dioxygenases (Dittmann et al., 2002; Mai et al., 2004; Xie et al., 2020). Therefore, it is speculated that *P. ostreatus* decomposes these aromatic intermediates (such as vanillic acid and protocatechuic acid) *via* the β-ketoadipate pathway.

In this study, *P. ostreatus* degraded lignin primarily during its mycelium growth with up to 54.04% lignin degradation. Its culture produces laccase and manganese dependent peroxidase, and no lignin peroxidase activity was detected. The results of nuclear magnetic resonance spectroscopy and Fourier transform infrared spectroscopy analyses of significant changes in lignin structure revealed that S lignin units were more degraded than G lignin units. The Gas Chromatography-Mass Spectrometer analysis of low-molecular-weight compounds revealed that the delignification resulted in the formation of alcohols, organic acids, benzodiazepines, and alkanes. These results demonstrate that S-type and G-type lignin monomers were the main products of lignin degradation by *P. ostreatus*, and that lignin with S-type monomers were degraded preferentially.

## Data availability statement

The raw data supporting the conclusions of this article will be made available by the authors, without undue reservation.

## Author contributions

GL conceptualized the idea for the study and performed most of experimental operations. YW and DY performed some of experimental operations and data analysis and led the writing of the manuscript. GL and HZ critically reviewed the data analysis and contributed substantially to the writing. PZ, GZ, and CL made key suggestions for improving the paper. All authors contributed to the article and approved the submitted version.

## Funding

This work was supported by the Key Research and Development Projects of Anhui Province (201904F06020025) and the Doctoral Matching Funds Project for Agricultural Research (340000211264005000407).

## Conflict of interest

The authors declare that the research was conducted in the absence of any commercial or financial relationships that could be construed as a potential conflict of interest.

## Publisher’s note

All claims expressed in this article are solely those of the authors and do not necessarily represent those of their affiliated organizations, or those of the publisher, the editors and the reviewers. Any product that may be evaluated in this article, or claim that may be made by its manufacturer, is not guaranteed or endorsed by the publisher.
